# Supplementary material for: Evolution of Interferon-Gamma Aptamer with Good Affinity and Analytical Utility by a Rational In Silico Base Mutagenesis Post-SELEX Strategy
Source: Molecules. 2022 Sep 5;27(17):5725. doi: 10.3390/molecules27175725 (PMC9457990; doi:10.3390/molecules27175725)
Supplement: Supplementary file 1 [file molecules-27-05725-s001.zip › molecules-1836468-supplementary.pdf]

Supplementary Materials

Evolution of Interferon-Gamma Aptamer with Good Affinity and Analytical Utility by a Rational In Silico Base Mutagenesis Post-SELEX Strategy

Lianhui Zhao [1], Qionglin Wang [2], Yingai Yin [1], Yan Yang [1], Huifang Cui [3] and Yiyang Dong [1,*]

[1] College of Life Science and Technology, Beijing University of Chemical Technology, Beijing 100029, China
[2] Henan Key Laboratory of Children's Genetics and Metabolic Diseases, Children's Hospital Affiliated to Zhengzhou University, Zhengzhou 450018, China
[3] College of Life Sciences, Zhengzhou University, Zhengzhou 450001, China
* Correspondence: yydong@mail.buct.edu.cn; Tel.: +86-010-64446260

Table S1. List of oligonucleotides.

DNA sequences	Sequence(5'→3')
M5-5	CCGCCCAAATCCCGAAGGGAAGAGTGTAATGACGTCAAACCAGA CACATTACACACGCA
MB-10	FAM-CGCGCGTGTAATGTGGCGCG-BHQ1
MB-15	FAM-CGCGCGTGTAATGTGTCTGGGCGCG-BHQ1
MB-20	FAM-CGCGCGTGTAATGTGTCTGGTTTGAGCGCG-BHQ1
MB-25	FAM-CGCGCGTGTAATGTGTCTGGTTTGACGTCAGCGCG-BHQ1

The underlined letters represent the complementary sequences.

Table S2. B1-4 and mutants with low free energy in the first round library.

Name	Sequence(5’→3’)	Dot-bracket information	ΔG (kcal/mol)	ZDOCK score
B 1-4	CCGCCCAAATCCCTAAGAGAAGACTGTAATGAC ATCAAACCAGACACACTACACACGCA	.......................(((..............)))................	-1.03	1246.537
M1-1	CCGCCCAAATCCCTAAGAGAAGACTGTAGTGAC ATCAAACCAGACACACTACACACGCA	.........................((((((...............))))))).......	-4.76	1253.540
M1-2	CCGCCCAAATCCCTAAGAGAAGACTGGAATGAC ATCAAACCAGACACACTACACACGCA	.......................((((..((....))..))))................	-4.38	1389.447
M1-3	CCGCCCAAATCCCTAAGAGAAGACTGTAATGAC ATCAAACCAGACACATTACACACGCA	.........................((((((...............))))))).......	-3.90	1387.888
M1-4	CCGCCCAAATCCCTAAGCGAAGACTGTAATGAC ATCAAACCAGACACACTACACACGCA	.(((............)))....(((..............)))................	-3.61	1307.033
M1-5	CCGCCCAAATCCCTAAGAGAAGTCTGTAATGACA TCAAACCAGACACACTACACACGCA	.....................(((((..............)))))..............	-3.49	1438.918
M1-6	CCGCCCAAATCCCTAAGAGAAGACTGTAATGAC ATCAAACCAGTCACACTACACACGCA	.....................(((((..............)))))..............	-3.49	1264.011
M1-7	CCGCCCAAATCCCTAAGAGAAGACTGTAATGAC ATCAAAACAGACACACTACACACGCA	.......................((((..((....))..))))................	-3.46	1362.160
M1-8	CCGCCCAAATCCCTAAGGGAAGACTGTAATGAC ATCAAACCAGACACACTACACACGCA	...(((..........)))....(((..............)))................	-3.38	1447.860

M1-9	CCGCCCAACTCCCTAAGAGAAGACTGTAATGAC ATCAAACCAGACACACTACACACGCA	........(((.....)))....(((..............)))................	-3.34	1410.515
M1-10	CCGCCCAAATCCCTAAGAGAAGACTGTATTGACA TCAAACCAGACACACTACACACGCA	.......................(((..(((....)))..)))................	-3.18	1288.798

The matched brackets represent base pairs, and dots represent unpaired bases.

Table S3. Mutants with low free energy in the second round library.

Name	Sequence(5'→3')	Dot-bracket information	ΔG (kcal/mol)	ZDOCK score
M2-1	CCGCCCAAATCCCTAAGGGAAGACTGTAGTGA CATCAAACCAGACACACTACACACGCA	...(((..........)))......((((((...............)))))).......	-7.11	1261.302
M2-2	CCGCCCAAATCCCTAAGAGAAGACTGGAATGA CATCAAACCAGTCACACTACACACGCA	.....................((((((..((....))..))))))..............	-6.84	1311.845
M2-3	CCGCCCAAATCCCTAAGAGAAGTCTGGAATGA CATCAAACCAGACACACTACACACGCA	.....................((((((..((....))..))))))..............	-6.84	1297.627
M2-4	CCGCCCAAATCCCTAAGGGAAGACTGGAATGA CATCAAACCAGACACACTACACACGCA	...(((..........)))....((((..((....))..))))................	-6.73	1229.231
M2-5	CCGCCCAACTCCCTAAGAGAAGACTGGAATGA CATCAAACCAGACACACTACACACGCA	........(((.....)))....((((..((....))..))))................	-6.69	1265.095
M2-6	CCGCCCAAATCCCTAAGGGAAGACTGTAATGA CATCAAACCAGACACATTACACACGCA	...(((..........)))......((((((...............)))))).......	-6.25	1485.757

M2-7	CCGCCCAACTCCCTAAGAGAAGACTGTAATGA CATCAAACCAGACACATTACACACGCA	........(((.....)))......((((((...............)))))).......	-6.21	1309.004
M2-8	CCGCCCAAATCCCTAAGCGAAGTCTGTAATGAC ATCAAACCAGACACACTACACACGCA	.(((............)))..(((((..............)))))..............	-6.07	1311.561
M2-9	CCGCCCAAATCCCTAAGAGAAGACTGGATTGA CATCAAACCAGACACACTACACACGCA	.......................((((.(((....))).))))................	-5.98	1310.280
M2-10	CCGCCCAAATCCCTAAGAGAAGTCTGTAATGAC ATCAAAACAGACACACTACACACGCA	.....................((((((..((....))..))))))..............	-5.92	1387.575

Table S4. Mutants with low free energy in the third round library.

Name	Sequence(5'→3')	Dot-bracket information	ΔG (kcal/mol)	ZDOCK score
M3-1	CCGCCCAAATCCCTAAGGGAAGACTGTAATG ACATCAAACCAGACACATTACAGACGCA	...(((..........)))....((((((((...............)))))))).....	-9.44	1370.869
M3-2	CCGCCCAAATCCCTAAGCGAAGACTGGAATG ACATCAAACCAGTCACACTACACACGCA	.(((............)))..((((((..((....))..))))))..............	-9.42	1295
M3-3	CCGCCCAAATCCCTAAGCGAAGTCTGGAATG ACATCAAACCAGACACACTACACACGCA	.(((............)))..((((((..((....))..))))))..............	-9.42	1292.590
M3-4	CCGCCCAAATCCCTAAGGGAAGAGTGTAATG ACATCAAACCAGACACATTACACACGCA	...(((..........)))....((((((((...............)))))))).....	-9.21	1385.598
M3-5	CCGCCCAAATCCCTAAGGGAAGACTGGAATG ACATCAAACCAGTCACACTACACACGCA	...(((..........)))..((((((..((....))..))))))..............	-9.19	1245.219

M3-6	CCGCCCAAATCCCTAAGTGAAGACTGGAATG ACATCAAACCAGTCACACTACACACGCA	...............((((..((((((..((....))..)))))).))))..........	-9.16	1460.141
M3-7	CCGCCCAAATCCCTAAGAGAAGACTGGAATG ACATCAAACCAGTCACTCTACACACGCA	...............((((..((((((..((....))..)))))).))))..........	-8.82	1214.207
M3-8	CCGCCCAAATCCCTAAGCGAAGTCTGTAATGA CATCAAAACAGACACACTACACACGCA	.(((............)))..((((((..((....))..)))))))..............	-8.50	1369.698
M3-9	CCGCCCAAATCCCTAAGCGAAGTCTGTAATGA CATCAAAACAGACACACTACACACGCA	.(((............)))..((((((..((....))..)))))))..............	-8.50	1243.932
M3-10	CCGCCCAAATCCCTAAGAGAAGACTGGATTG ACATCAAACCAGTCACACTACACACGCA	.....................((((((.(((....))).))))))..............	-8.44	1270.633

Table S5. Mutants with low free energy in the fourth round library.

Name	Sequence(5’→3’)	Dot-bracket information	ΔG (kcal/mol)	ZDOCK score
M4-1	CCGCCCAAATCCCTAAGGGCAGACTGTAATG ACATCAAACCAGACACATTACAGACGCA	..((((..........))))...((((((((...............))))))))....	-11.42	1274.997
M4-2	CCGCCCAAATCCCTAAGGGAAGACTGTAAT GACGTCAAACCAGACACATTACAGACGCA	...(((..........)))....((((((((..(((......))).))))))))....	-11.41	1293.240
M4-3	CCGCCCAAATCCCGAAGGGAAGACTGTAAT GACATCAAACCAGACACATTACAGACGCA	.........((((...))))...((((((((...............))))))))....	-11.24	1321.559
M4-4	CCGCCCAAATCCCTAAGGGAAGTGTGTAATG ACATCAAACCAGACACATTACACACGCA	...(((..........)))..((((((((((...............))))))))))...	-11.35	1246.058

M4-5	CCGCCCAAATCCCTAAGGGCAGAGTGTAAT GACATCAAACCAGACACATTACACACGCA	..((((..........))))...((((((((...............))))))))).....	-11.19	1235.186
M4-6	CCGCCCAAATCCCTAAGGGAAGAGTGTAAT GACGTCAAACCAGACACATTACACACGCA	...(((..........)))....((((((((..(((......))).))))))))).....	-11.18	1341.062
M4-7	CCGCCCAAATCCCTAAGGGAAGACTGTAAT GACATCAAACCAGACACATTACAGTCGCA	...(((..........)))..((((((((((...............))))))))))...	-11.01	1313.665
M4-8	CCGCCCAAATCCCGAAGGGAAGAGTGTAAT GACATCAAACCAGACACATTACACACGCA	.........((((...))))...((((((((...............))))))))).....	-11.01	1296.120
M4-9	CCGCCCAAATCCCTAAGGGAAGAGTGTAAT GACATCAAACCAGACACATTACACTCGCA	...(((..........)))..((((((((((...............))))))))))...	-11.01	1321.879
M4-10	CCGCCCAAATCCCTAAGTGAAGACTGGATT GACATCAAACCAGTCACACTACACACGCA	...............((((..((((((.(((....))).)))))).))))..........	-10.76	1354.180

Table S6. Mutants with low free energy in the fifth round library.

Name	Sequence(5'→3')	Dot-bracket information	ΔG (kcal/mol)	ZDOCK score
M5-1	CCGCCCAAATCCCTAAGTGAAGACTGGATTG ACATCAATCCAGTCACACTACACACGCA	...............((((..((((((((((....)))))))))).))))..........	-14.13	1368.866
M5-2	CCGCCCAAATCCCGAAGGGAAGACTGTAATG ACGTCAAACCAGACACATTACAGACGCA	.........((((...))))...((((((((..(((......))).))))))))).....	-13.21	1202.775
M5-3	CCGCCCAAATCCCTAAGGGCAGAGTGTAATG ACGTCAAACCAGACACATTACACACGCA	..((((..........))))...((((((((..(((......))).))))))))).....	-13.16	1433.854

M5-4	CCGCCCAAATCCCTAAGGGACGAGTGTAATG ACATCAAACCAGACACATTACACTCGCA	...(((..........))).(((((((((((...............)))))))))))..	-13.01	1261.402
M5-5	CCGCCCAAATCCCGAAGGGAAGAGTGTAATG ACGTCAAACCAGACACATTACACACGCA	.........((((...))))...((((((((..(((......))).))))))))).....	-12.98	1543.334
M5-6	CCGCCCAAATCCCGAAGGGAAGAGTGTAATG ACATCAAACCAGACACATTACACTCGCA	.........((((...)))).((((((((((...............))))))))))...	-12.78	1320.181
M5-7	CCGCCCAAATCCCGAAGGGAAGTCTGTAATG ACATCAAACCAGACACATTACAGACGCA	.........((((...)))).((((((((((...............))))))))))...	-12.78	1398.095
M5-8	CCGCCCAAATCCCGAAGGGAAGACTGTAATG ACATCAAACCAGACACATTACAGTCGCA	.........((((...)))).((((((((((...............))))))))))...	-12.78	1460.231
M5-9	CCGCCCAAATCCCTAAGGGAAGAGTGTAATG ACGTCAAACCAGACACATTACACTCGCA	...(((..........)))..((((((((((..(((......))).))))))))))...	-12.75	1441.206
M5-10	CCGCCCAAATCCCGAAGGGATGACTGTAATG ACATCAAACCAGACACATTACAGACGCA	........(((((...)))))..((((((((...............)))))))).....	-12.46	1305.417

Table S7. Mutants with low free energy in the sixth round library.

Name	Sequence(5’→3’)	Dot-bracket information	ΔG (kcal/mol)	ZDOCK score
M6-1	CCGCCCAAATCCCTAAGGGCGGAGTGTAATG ACGTCAAACCAGACACATTACACACGCA	((((((..........)))))).((((((((..(((......))).))))))))).....	-16.29	1314.663
M6-2	CCGCCCAAATCCCGAAGGGAAGTGTGTAATG ACGTCAAACCAGACACATTACACACGCA	.........((((...)))).((((((((((..(((......))).))))))))))...	-15.09	1406.824

M6-3	CCGCCCAAATCCCTAAGGGCAGAGTGTAATG ACGTCAAACCAGACGCATTACACACGCA	..((((..........))))...((((((((.((((......))))))))))))).....	-15.08	1343.275
M6-4	CCGCCCAAATCCCTAAGGGACGAGTGTAATG ACGTCAAACCAGACACATTACACTCGCA	...(((..........))).(((((((((((..(((......))).)))))))))))..	-14.98	1380.029
M5-5	CTGCCCAAATCCCTAAGGGCAGAGTGTAATG ACGTCAAACCAGACACATTACACACGCA	((((((..........)))))).((((((((..(((......))).))))))))).....	-14.97	1241.493
M6-6	CCGCCCAAATCCCGAAGGGAAGAGTGTAATG ACGTCAAACCAGACGCATTACACACGCA	.........((((...))))...((((((((.((((......))))))))))))).....	-14.90	1394.455
M6-7	CCGCCCAAATCCCTAAGGGAAGAGTGTAATG ACGTCAAACCAGACGCATTACACTCGCA	...(((..........)))..((((((((((.((((......))))))))))))))))...	-14.90	1354.663
M6-8	CCGCCCAAATCCCGAAGGGAAGAGTGTAATG ACGTCAAACCAGACACATTACACTCGCA	.........((((...)))).((((((((((..(((......))).))))))))))...	-14.75	1307.655
M6-9	CCGCCCAAATCCCTAAGGGCAGTGTGTAATGA CGTCAAACCAGACACATTACACACGCA	..((((..........)))).((((((((((..(((......))).))))))))))...	-14.70	1381.942
M6-10	CCGCCCAAATCCCGAAGGGACGACTGTAATG ACATCAAACCAGACACATTACAGTCGCA	.........((((...))))(((((((((((...............)))))))))))..	-14.57	1331.607

Table S8. The binding residues of B1-4 and M5-5 based on the Mfold prediction interacted with IFN-γ through hydrogen bonding.

Sequence	Binding residues
B 1-4	^{I}Tyr14-C54, ^{II}Asn25-A34, ^{I}Lys58-A32, ^{I}Lys61-G31, ^{I}Lys94-T35, ^{I}Asn97-C33, ^{I}Tyr98-A34, ^{I}Ser99-C33, ^{II}Thr101-C54, ^{I}Asp102-A34, ^{II}Gln106-C54
M 5-5	^{I}Tyr14-C12, ^{I}Tyr14-A20, ^{I}Asn59-A9, ^{II}Asn59-C2, ^{II}Asn59-G3, ^{I}Asp63-C11, ^{I}Ser65-G14, ^{II}Thr101-A9, ^{I}Gln106-C2, ^{II}Gln106-T10

I, II representative the two monomers of IFN-γ, respectiviely.

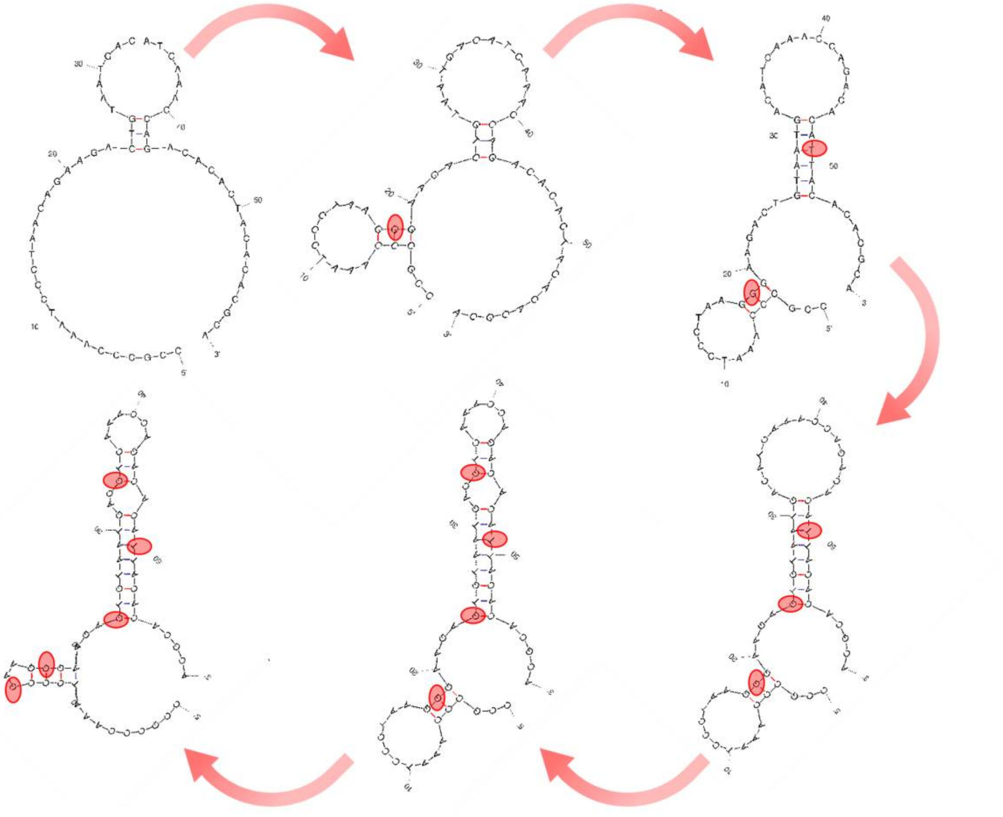

Figure S1. The phylogeny of the M5-5 aptamer predicted by the Mfold. The mutation sites are shaded in red.

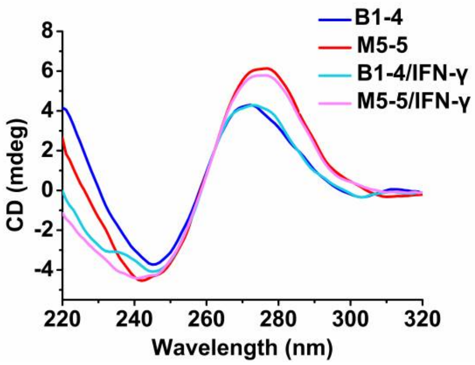

Figure S2. Circular dichroism (CD) analysis of B1-4 and M5-5 with or without IFN-γ.

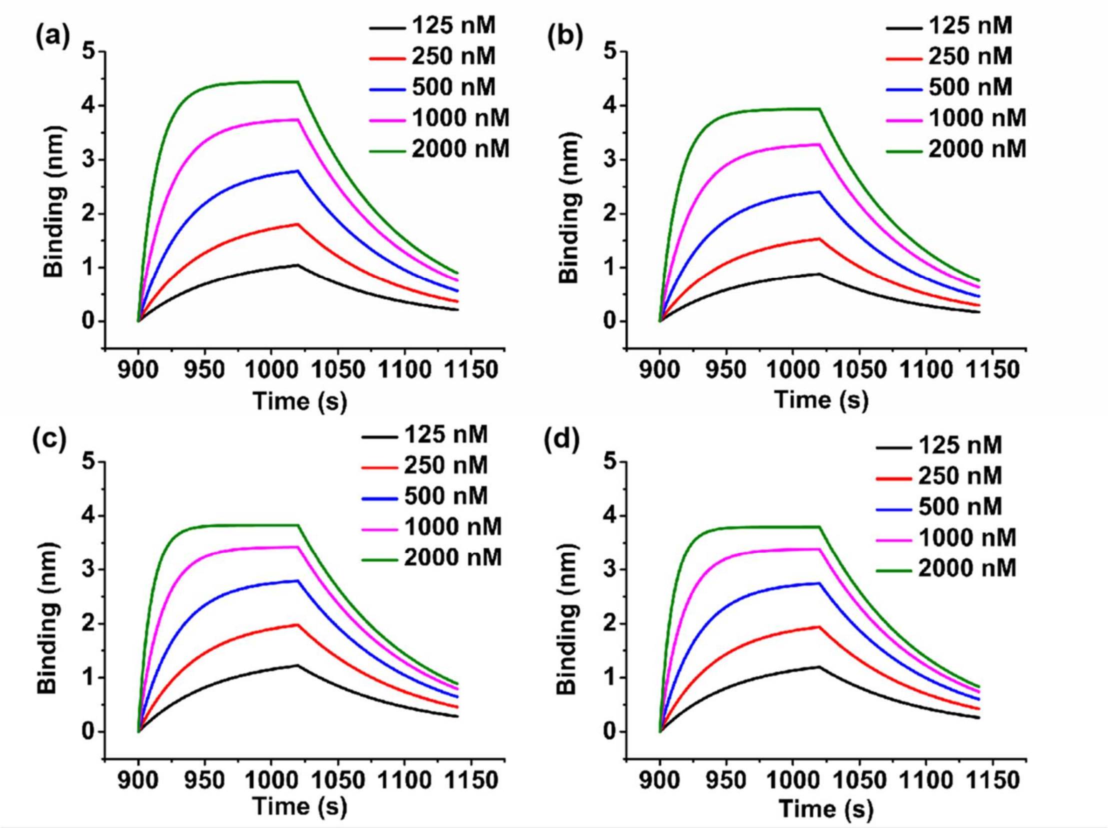

Figure S3. BLI characterization of the affinity of (a) M1-8, (b) M2-6, (c) M3-6 and (d) M4-10.

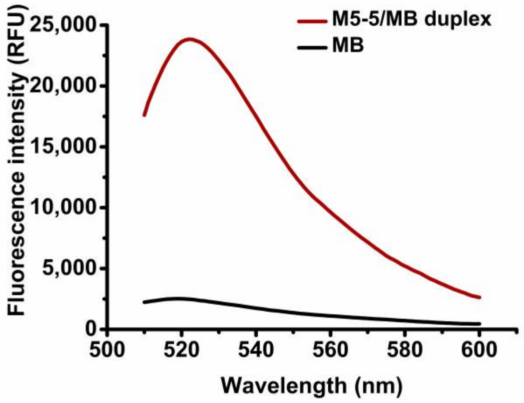

Figure S4. Fluorescence emission spectra of the M5-5/MB duplex and MB.
